# Gastroesophageal reflux disease increases predisposition to severe COVID‐19: Insights from integrated Mendelian randomization and genetic analysis

**DOI:** 10.1111/ahg.12584

**Published:** 2024-11-12

**Authors:** Jingjing Pan, Jianhua Li

**Affiliations:** ^1^ Department of Microbiology Zhejiang Provincial Centers for Disease Control and Prevention Hangzhou China; ^2^ Zhejiang Key Laboratory of Public Health Detection and Pathogenesis Research Hangzhou China

**Keywords:** colocalization analysis, COVID‐19, gastroesophageal reflux disease, Mendelian randomization, molecular pathway analysis, shared genomic loci

## Abstract

**Objective:**

This study aims to investigate the potential causal relationship, shared genomic loci, as well as potential molecular pathways and tissue‐specific expression patterns between gastroesophageal reflux disease (GERD) and the risk of hospitalized/severe 2019 coronavirus disease (COVID‐19).

**Methods:**

We employed linkage disequilibrium score regression and bidirectional Mendelian randomization (MR) analysis to explore potential genetic associations between GERD (*N* = 602,604) and hospitalized COVID‐19 (*N* = 2095,324) as well as severe COVID‐19 (*N* = 1086,211). Additionally, shared genomic loci were extracted from common pivotal regions, further confirmed through corresponding colocalization analyses. GERD‐driven molecular pathway network was constructed using extensive literature data mining to understand the molecular‐level impacts of GERD on COVID‐19.

**Results:**

Our results revealed a significant positive genetic correlation between GERD and both hospitalized (*r_g_*  =  0.418) and severe COVID‐19 (*r_g_*  =  0.314). Furthermore, the MR analysis demonstrated a unidirectional causal effect of genetic predisposition to GERD on COVID‐19 outcomes, including hospitalized COVID‐19 (odds ratio [OR]: 1.33, 95% confidence interval [CI]: 1.27–1.44, *p* = 9.17e − 12) and severe COVID‐19 (OR: 1.27, 95% CI: 1.18–1.37, *p* = 1.20e − 05). Additionally, GERD and both COVID‐19 conditions shared one genomic locus with lead‐SNPs rs1011407 and rs1123573, corresponding to the transcription factor BCL11A. Colocalization analysis further demonstrated a significant positive correlation between genome‐wide association study and expression quantitative trait locus (eQTL) abnormalities, including rs1011407 (eQTL_*p* = 2.35e − 07) and rs1123573 (eQTL_*p* = 2.74e − 05). Molecular pathway analysis indicated that GERD might promote the progression of COVID‐19 by inducting immune‐activated and inflammation‐related pathways.

**Conclusion:**

These findings confirm that genetically determined GERD may increase the susceptibility to hospitalized/severe COVID‐19. The shared genetic loci and the potential molecular pathways offer valuable insights into causal connections between GERD and COVID‐19.

## 1 BACKGROUND

The COVID‐19 pandemic, stemming from the novel coronavirus SARS‐CoV‐2, has exerted profound global repercussions (Chow et al., 2023; Hu et al., 2021; Kaul et al., 2021). This viral pathogen gains entry into host cells via two key components: transmembrane serine protease 2 (TMPRSS2) and the ACE‐II cell receptor (ACE2) (Saengsiwaritt et al., 2022; Senapati et al., 2021). Infected individuals may face the risk of excessive immune response and pneumonia, and a lack of timely treatment may lead to fatal consequences (Trougakos et al., 2021). Notwithstanding substantial strides in vaccine development, there remains a pressing need for comprehensive investigation into the factors contributing to COVID‐19 susceptibility (Fiolet et al., 2022). Furthermore, the omnipresent global pandemic continues to loom menacingly, with a multitude of risk factors and disease incidents intertwined with COVID‐19 (Zhang et al., 2023). Among these factors, gastrointestinal disorders have emerged as significant determinants of both disease severity and mortality, a connection that has garnered extensive discourse within scholarly circles (Cao et al., 2021).

The susceptivity association between gastroesophageal reflux disease (GERD) and the severe manifestation of COVID‐19 has been widely acknowledged since the onset of the pandemic (Ong, An, et al., 2022). GERD, a chronic digestive ailment, is characterized by the frequent reflux of stomach acid into the esophagus (Maret‐Ouda et al., 2020). Notably, there exist shared symptoms between GERD and COVID‐19, such as chest pain, which can introduce complexities in diagnosing and treating these conditions (He et al., 2022). Moreover, the lifestyle, psychological well‐being, and medication usage of individuals during the COVID‐19 pandemic could indirectly establish connections between the two maladies (Garg et al., 2022). The COVID‐19 pandemic and lockdown have been reported to exacerbate GERD symptoms, with a large‐scale retrospective study confirming an increased morbidity rate of GERD in COVID‐19 patients within 1 year of infection (Al‐Momani, Balawi, et al., 2023; Xu et al., 2023). Additionally, the prominent COVID‐19 receptors ACE2 and TMPRSS2 are co‐expressed in both the epithelial and glandular cells of the esophagus, potentially serving as a crucial factor linking GERD and COVID‐19 (Al‐Momani, Mashal, et al., 2023). Previous studies have demonstrated that multiple esophageal cells could produce proinflammatory cytokines like IL‐1β and IL‐6, contributing to esophagitis and motor abnormalities (Rieder et al., 2007). These proinflammatory cytokines might participate in the pathogenesis of cytokine storms in severe COVID‐19, supporting the potentially increased susceptibility of GERD patients to severe COVID‐19 (Ye et al., 2020). In sum, although GERD and COVID‐19 are distinct conditions, instances arise wherein they exhibit overlapping or interrelated symptoms and susceptivity associations.

Nevertheless, a majority of these studies were observational and could not further establish potential causal links between GERD and COVID‐19. Previous large‐scale genome‐wide association study (GWAS) datasets provide an intriguing avenue to validate these susceptible associations in both COVID‐19 and GERD. Mendelian randomization (MR) analysis has been widely used to investigate potential causal effects of various comorbidities on COVID‐19, such as neurodegenerative disorders (Moneta et al., 1987), genetic obesity (Aung et al., 2020), multiple sclerosis (Baranova, Cao, Teng, et al., 2023), and others. To date, only one MR analysis explored the genetic relationship between GERD and COVID‐19, reporting odds ratio (OR) values of 1.15 (Ong, Gharahkhani, et al., 2022). However, this study did not delve into shared genomic loci and potential molecular pathways underlying their connections.

In this study, we performed a bidirectional two‐sample MR analysis to investigate the potential causal relationships between GERD comorbidity and hospitalized/severe COVID‐19. Moreover, shared genomic loci were extracted from common pivotal regions reported for each trait, and corresponding colocalization analysis was conducted to explore potential GWAS–expression quantitative trait locus (GWAS–eQTL) effects. Finally, we utilized the knowledge‐based network analysis to identify potential molecular pathways and tissue‐specific expression patterns related to this connection.

## 2 METHODS

### 2.1 Study design

**FIGURE 1:**
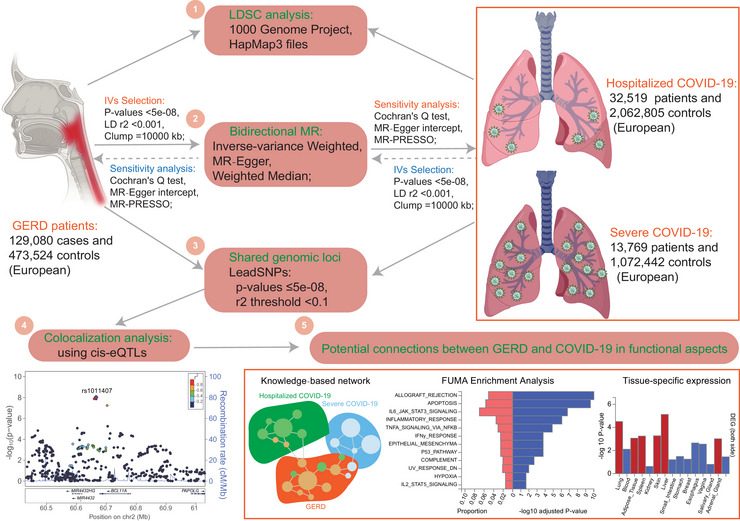
**The flowchart of the analyses performed in this study**. The design of our study and the workflow of the selection of instrumental variants and analytical methods. eQTLs, expression quantitative trait loci; IVs, instrumental variants; LDSC, linkage disequilibrium score regression.

**Figure** **1** summarizes the design of our study and the workflow of the selection of instrumental variants (IVs) and analytical methods. This included linkage disequilibrium score regression (LDSC) analysis, bidirectional MR analysis, shared genomic locus analysis, colocalization analysis, and functional connection evaluation. The details of analytical methods are described as follows.

### 2.2 GWAS dataset preparation

The MR analysis leveraged GWAS summary datasets to assess the potential association between GERD and COVID‐19. The GWAS summary results of COVID‐19 patients were obtained from the COVID‐19 Host Genetics Initiative (HGI) database round 7 (https://www.covid19hg.org/results/r7/) (Initiative, 2020), including 32,519 hospitalized patients and 2062,805 healthy controls, as well as 13,769 severe cases and 1072,442 controls. The HGI database is currently recognized as the largest GWAS database for COVID‐19 patients. All these participants were of European descent. The GWAS summary statistics for GERD cohorts were retrieved from the GWAS Catalog database (https://www.ebi.ac.uk/gwas/home, trait ID: GCST90000514) (Ong, An, et al., 2022), including 129,080 cases and 473,524 controls from the European populations.

### 2.3 Linkage disequilibrium score regression (LDSC) analysis

To explore the genetic correlation between GERD and COVID‐19 outcomes, we performed the LD score regression analysis using the ldscr package (https://github.com/mglev1n/ldscr). The 1000 Genome Project Phase 3 was utilized to elevate the LD structures in European populations (Bulik‐Sullivan et al., 2015). Based on single‐nucleotide polymorphisms (SNPs) from the 1000 Genomes and HapMap3 files, we determined significant genetic correlations by false discovery rate <0.05.

### 2.4 MR analysis and sensitivity analysis

We utilized a bidirectional two‐sample MR analysis to investigate the potential causal relationships between GERD and hospitalized/severe COVID‐19. IVs were derived from significant SNPs in GERD datasets with *p*‐values <5e − 08, and *r* ^2^ <0.01 with clumping window size <10,000 kb. Interferential IVs were excluded with allele frequencies close to 0.5 and *p*‐values <0.01 in the COVID‐19 datasets. To ensure the robustness of IVs for exposures, we calculated the *F* statistic according to the following formula: $F=\frac{R^{2}}{1-R^{2}}\times \frac{N-K-1}{K}$, where *R* ^2^ represents the total variance of exposures explained by selected SNPs, *N* represents the sample size, and *K* represents the number of chosen IVs. The SNPs with *F*‐value ≥10 were considered to have adequate strength, ameliorating the limitations of weak instrument bias in the two‐sample MR method.

The primary evaluation of MR results was conducted using the inverse‐variance weighted (IVW) method, complemented with estimations of the MR Egger regression (MR‐Egger) and weighted median (WM) methods using the TwoSampleMR package (https://github.com/MRCIEU/TwoSampleMR) (Burgess et al., 2013). For sensitivity analysis, we assessed the heterogeneity by using Cochran's *Q* test (*Q*_*p* value >0.05) (Kulinskaya & Hoaglin, 2023) and examined the average horizontal pleiotropy using the intercept from MR‐Egger (Bao et al., 2023) and MR‐Pleiotropy Residual Sum and Outlier (MR‐PRESSO) test (Verbanck et al., 2018) (Global Test_p >0.05).

### 2.5 Shared genomic loci analysis and colocalization analysis

To further identify shared risk genes between GERD and hospitalized/severe COVID‐19, we overlapped common genomic loci from the GWAS summary datasets of both diseases. Initially, we employed the Functional Mapping and Annotation (FUMA) tool to screen LD‐independent genomic loci and to map SNPs to their corresponding genes (Watanabe et al., 2017). Independent significant SNPs (IndSigSNPs) were defined as *p*‐values ≤5e − 08 and *r* ^2^ threshold <0.6, whereas the lead SNPs were further identified as a subset of IndSigSNPs with *r* ^2^ <0.1 within a 500 kb radius. Subsequently, we determined the genomic risk loci by consolidating lead SNPs located within 500 kb of each other and conducted the clumping procedures based on the “1000G Phase 3 EUR” reference panel. Visualization of the regional distribution of shared genomic loci was generated by the LocusZoom software (http://locuszoom.org/) (Pruim et al., 2010). To validate whether the shared genomic loci influenced disease phenotypes by altering gene expression, we performed the colocalization analysis based on shared lead SNPs and publicly available *cis*‐eQTLs using the coloc package (Wang et al., 2020). The locuscomparer package was applied to visualize the results of GWAS–eQTL colocalization (Liu et al., 2019). To evaluate the expressional levels of genes corresponding to shared genomic loci, we obtained RNA‐seq datasets of COVID‐19 patients from Gene Expression Omnibus (GEO) database (ID: GSE157103, https://www.ncbi.nlm.nih.gov/geo/query/acc.cgi?acc=GSE157103), which included data from 100 COVID‐19 patients and 26 controls (Overmyer et al., 2021).

### 2.6 Knowledge‐based network analysis

To investigate the potential etiological links between GERD and COVID‐19 at the molecular level, we constructed a network of molecular pathways connecting these two conditions through extensive literature data mining. The connection was generated using an AI‐powered tool for literature‐data mining developed by AIC LLC (http://gousinfo.com/cn/index.html), and the network was visualized by Cytoscape v3.10.0 software (Shannon et al., 2003). Protein–protein interaction (PPI) network among these regulatory genes was derived from the STRING v11.0 database (Szklarczyk et al., 2023) and constructed using Cytoscape software. The functional enrichment analysis of regulatory genes was annotated by hallmark gene sets (MsigDB) through FUMA tools. The tissue‐specific expression pattern of these genes was identified by gene‐property analyses comparing tissue‐specific expression data from GTEx V8 tissues with GWAS hits.

### 2.7 Ethics statement

All GWAS summary statistics used in this study were derived from previously published studies, which received approval from corresponding ethical committees and obtained the informed consent forms from all participants.

## 3 RESULTS

### 3.1 Genetic correlation between GERD and COVID‐19

Genetic correlation analyses revealed that GERD exhibited a significant positive genetic correlation with hospitalized COVID‐19 (*r*_g_  =  0.418 ± 0.0329, *p*  =  5.31e − 37) and severe COVID‐19, respectively (*r*_g_  =  0.314 ± 0.0313, *p*  =  1.19e − 23) (Table 1).

**TABLE 1:** Genetic correlations between gastroesophageal reflux disease (GERD) and hospitalized/severe COVID‐19 outcomes.

Trait 1	Trait 2	Mean_chisq	Lambda_GC	Intercept	*r*_g_	SE	*p*‐value
GERD	Hospitalized COVID‐19	1.166	1.102	1.007	0.418	0.0329	5.31e − 37
GERD	Severe COVID‐19	1.162	1.095	1.001	0.314	0.0313	1.19e − 23

Abbreviations: chisq, chi‐square; *r*_g_, genetic correlation coefficient; SE, standard error.

### 3.2 Causal effect assessment by MR analysis

**FIGURE 2:**
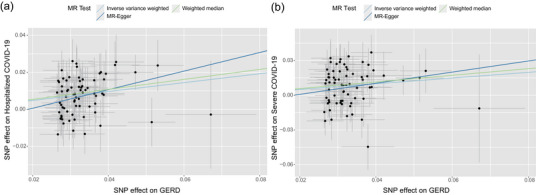
**The causal effect of gastroesophageal reflux disease (GERD) on COVID‐19 outcomes: (a and b)** The Mendelian randomization (MR) analysis results of GERD and hospitalized COVID‐19 (a) and severe COVID‐19 (b). The x‐ray represents the single‐nucleotide polymorphism (SNP) effects on GERD, and the y‐ray represents the SNP effects on COVID‐19 outcomes. Each point represents an instrumental variable, and lines represent effect values (*β*) of exposures on outcomes. IVW, inverse variance weighted (multiplicative random effects); MR‐Egger, Mendelian randomization Egger regression; WM, weighted median.

We, respectively, screened 74 and 72 IVs from GERD datasets for hospitalized COVID‐19 and severe COVID‐19 traits and performed MR analysis by integrating the IVW, MR‐Egger, and WM methods. The results demonstrated that a genetic predisposition to GERD has a causal impact on COVID‐19 outcomes, including hospitalized COVID‐19 (OR: 1.33, 95% CI: 1.27–1.44, *p* = 9.17e − 12) and severe COVID‐19 (OR: 1.27, 95% CI: 1.18–1.37, *p* = 1.20e − 05) (Figure 2; Table 2).

**TABLE 2:** Causal effects of gastroesophageal reflux disease (GERD) on hospitalized and severe COVID‐19.

Exposure	Outcome	Methods	nSNP	*β*	SE	OR [95% CI]	*p*‐value	*Q*_*p*‐value	*P*_test *p*‐value
**GERD**	**Hospital COVID‐19**	IVW	74	0.240	0.035	1.33 [1.27–1.44]	**9.17e** − **12**	0.875	NA
**GERD**	**Hospital COVID‐19**	MR‐Egger	74	0.502	0.243	2.20 [1.41–3.42]	**0.043**	0.881	0.280
**GERD**	**Hospital COVID‐19**	WM	74	0.270	0.058	1.36 [1.22–1.51]	**3.79e** − **06**	NA	NA
**GERD**	**Hospital COVID‐19**	MR‐PRESSO	74	NA	NA	NA	0.886	NA	NA
**Hospital COVID‐19**	**GERD**	IVW	9	0.025	0.027	0.97 [0.93–1.03]	0.337	0.182	NA
**Hospital COVID‐19**	**GERD**	MR‐Egger	9	0.022	0.080	1.02 [0.87–1.20]	0.788	0.150	0.546
**Hospital COVID‐19**	**GERD**	WM	9	0.016	0.029	0.98 [0.93–1.04]	0.589	NA	NA
**Hospital COVID‐19**	**GERD**	MR‐PRESSO	9	NA	NA	NA	0.525	NA	NA
**GERD**	**Severe COVID‐19**	IVW	72	0.247	0.056	1.27 [1.18–1.37]	**1.20e** − **05**	0.257	NA
**GERD**	**Severe COVID‐19**	MR‐Egger	72	0.481	0.366	1.65 [1.03–2.66]	0.194	0.242	0.521
**GERD**	**Severe COVID‐19**	WM	72	0.287	0.082	1.31 [1.17–1.46]	**4.60e** − **04**	NA	NA
**GERD**	**Severe COVID‐19**	MR‐PRESSO	72	NA	NA	NA	0.286	NA	NA
**Severe COVID‐19**	**GERD**	IVW	11	0.016	0.010	1.02 [1.00–1.04]	0.089	0.820	NA
**Severe COVID‐19**	**GERD**	MR‐Egger	11	0.011	0.015	1.01 [0.98–1.04]	0.458	0.765	0.673
**Severe COVID‐19**	**GERD**	WM	11	0.016	0.012	1.02 [0.99–1.04]	0.169	NA	NA
**Severe COVID‐19**	**GERD**	MR‐PRESSO	11	NA	NA	NA	0.864	NA	NA

Abbreviations: CI, confidence interval; IVW, inverse variance weighted (multiplicative random effects); MR‐Egger, Mendelian Randomization Egger regression; MR‐PRESSO, Mendelian Randomization Pleiotropy Residual Sum and Outlier; nSNP, number of single‐nucleotide polymorphism; OR, odds ratio; P_test, pleiotropy_test; SE, standard error; WM, weighted median.

Our sensitivity analyses eliminated both heterogeneity and horizontal pleiotropy in the MR analysis. The Cochran *Q* test did not support the existence of heterogeneity in both hospitalized (*Q*_*p* = 0.875) and severe COVID‐19 (*Q*_*p* = 0.257). Additionally, the MR‐PRESSO and pleiotropy tests also did not support the existence of horizontal pleiotropy among the selected IVs in the MR process, including hospitalized (pleiotropy test *p *= 0.280, Global test *p* = 0.886) and severe COVID‐19 (pleiotropy test *p *= 0.521, Global test *p* = 0.286) (Table 2). Furthermore, we conducted reversed MR analysis using COVID‐19 traits as exposures and GERD as outcomes. This analysis revealed no causal effects of genetic predisposition to hospitalized (OR: 0.97, 95% CI: 0.93–1.03, *p* = 0.337) and severe COVID‐19 (OR: 1.02, 95% CI: 1.00–1.04, *p* = 0.089) on GERD traits (Table 2).

### 3.3 Identification of shared genomic loci and colocalization analysis

**FIGURE 3:**
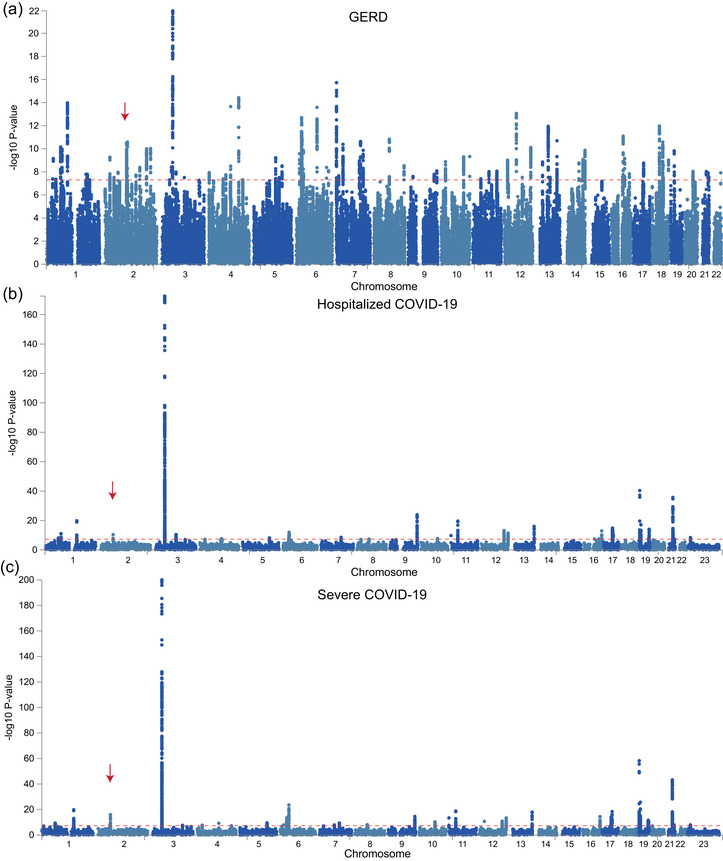
**Manhattan plot showing results of genome‐wide association study (GWAS) in gastroesophageal reflux disease (GERD) and hospitalized/severe COVID‐19 traits**. The x‐ray represents positions of single‐nucleotide polymorphisms (SNPs) in 22 chromosomes, and the y‐ray represents the −log10(*p*‐values) of each SNP. The threshold of *p*‐values was set as <5e − 8 (red lines). The red arrow shows the shared genomic loci between GERD and two COVID‐19 conditions.

**FIGURE 4:**
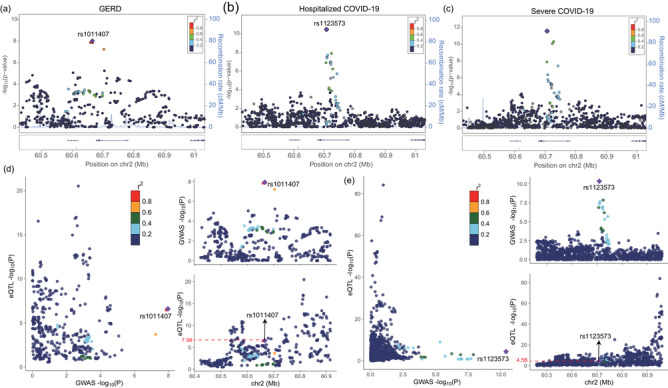
**Shared genomic loci and colocalization analysis: (a–c)** The location of shared genomic loci in chr2 between gastroesophageal reflux disease (GERD) (rs1011407) and hospitalized/severe COVID‐19 (rs1123573). Both two single‐nucleotide polymorphisms (SNPs) were located in regions of BCL11A; **(d and e)** colocalization analysis showing significant positive correlation between genome‐wide association study (GWAS) and expression quantitative trait loci (eQTL) in both rs1011407 (eQTL *p* = 2.35e − 07) and rs1123573 (eQTL *p *= 2.74e − 05).

FUMA analysis successfully identified a total of 88, 30, and 31 genomic loci associated with GERD, hospitalized, and severe COVID‐19, respectively (Figure 3). Interestingly, only one shared genomic locus was overlapped between GERD (rs1011407) and two COVID‐19 (rs1123573), including regions located within chr2p16.1 (Table 3). By coincidence, these two SNPs correspond to the transcription factor BCL11A (Figure 4a–c). Moreover, the colocalization analysis further demonstrated significant positive correlation between GWAS and eQTL abnormalities, including rs1011407 (eQTL *p*‐value = 2.35e − 07) and rs1123573 (eQTL *p*‐value = 2.74e − 05) (Figure 4d,e). These results indicated shared SNPs in both GERD and COVID‐19 significantly influence gene expressions via eQTL effects. The expression of *BCL11A* was significantly decreased in severe cases compared to mild status for both COVID‐19 and controls. Notably, in severe cases, COVID‐19 patients exhibited lower *BCL11A* expression than controls, suggesting that *BCL11A* expression was associated with disease severity in COVID‐19 patients (Figure S1).

**TABLE 3:** Shared genomic loci among gastroesophageal reflux disease (GERD), hospitalized COVID‐19, and severe COVID‐19.

Trait	SNP	CHR	BP	Start:End	A1/A2	*p*‐value	Genes
**GERD**	rs1011407	2	60665768	60607636:60723905	A/G	1.09e − 08	BCL11A
**Hospital COVID‐19**	rs1123573	2	60707588	60705232:60727416	A/G	4.13e − 11	BCL11A
**Severe COVID‐19**	rs1123573	2	60707588	60705232:60751695	A/G	2.80e − 12	BCL11A

Abbreviations: BP, base pair; CHR, chromosome; SNP, single‐nucleotide polymorphism.

### 3.4 Molecular network of GERD and COVID‐19 by knowledge‐based analysis

**FIGURE 5:**
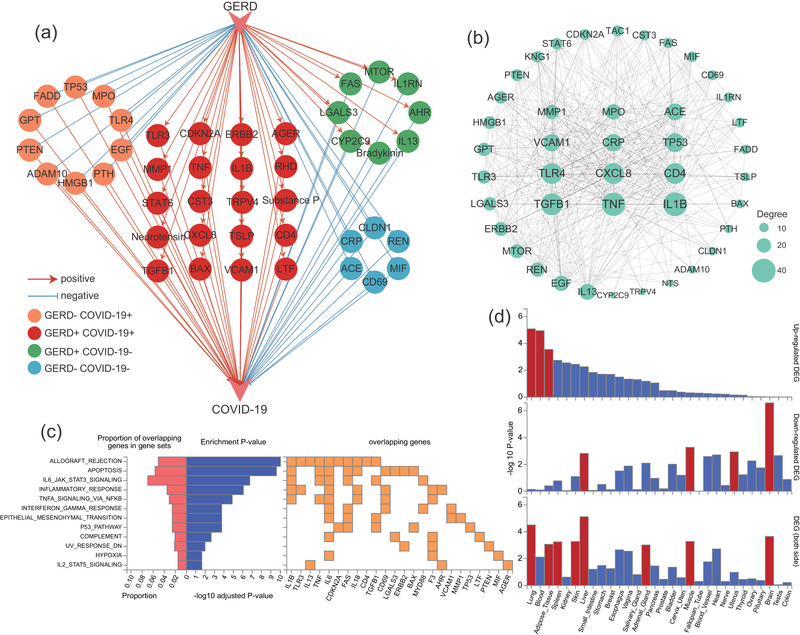
**Knowledge‐based pathway analysis and tissue‐based expressional analysis. (a)** Gastroesophageal reflux disease (GERD)‐driven molecular pathway influencing COVID‐19 by 44 regulatory genes, including 28 positive and 16 negative molecules. The red arrow represents promoting roles, and blue arrow represents inhibitory roles. **(b)** Protein–protein interaction (PPI) network among the regulatory genes showing tight interconnections. The size of nodes represents the number of interconnection with other nodes. **(c)** Hallmark‐based pathway enrichment analysis indicated that 44 regulatory genes were significantly enriched in immune‐activated and inflammation‐related pathways. **(d)** Tissue‐based expressional analysis revealed that 44 regulatory genes were upregulated in lung, blood, and adipose tissue while downregulating in liver, muscle, and brain tissues. The red columns represent significantly enriched differentially expressed gene (DEG) sets (false discovery rate [FDR] <0.05) in specific tissues.

Literature‐based pathway analysis identified 44 regulatory genes connecting GERD and COVID‐19, including 28 molecules positively associated with the pathophysiological processes of COVID‐19 and 16 regulators negatively influencing these processes (Figure 5a). The PPI network further validated the tight interconnections among these mediating proteins, highlighting the potential molecular regulatory role of GERD on COVID‐19 (Figure 5b). Moreover, hallmark‐based pathway enrichment analysis indicated that these regulatory genes were significantly enriched in immune‐activated and inflammation‐related pathways, including IL‐6–JAK–STAT3 signaling, inflammatory response, TNFA signaling via NFκB, and interferon‐gamma response (Figure 5c). Tissue‐based expressional analysis revealed that 44 regulatory genes were upregulated in lung, blood, and adipose tissue while downregulating in liver, muscle, and brain tissues (Figure 5d).

## 4 DISCUSSION

Gastrointestinal disorder is one of the most common comorbidities of COVID‐19. Previous epidemiological studies have indicated similar GERD‐related symptoms and common risk factors during the acute phase of COVID‐19 (Grant et al., 2020; Xu et al., 2023). However, the observational clinical studies cannot affirm causality in potential associations between GERD and COVID‐19. In this study, our genetic correlation analysis successfully validated the positive correlations between GERD and hospitalized/severe COVID‐19. Moreover, our MR analysis further identified potential causal impacts of GERD on hospitalized and severe COVID‐19, with OR values of 1.33 and 1.27, respectively. In contrast, the bidirectional MR analysis showed that two COVID‐19 statuses did not have causal effects on GERD disease (*p* > 0.05), indicating that GERD had a unidirectional genetic risk to COVID‐19. These results affirmed extensive evidence that gastrointestinal disorder may increase the susceptibility of COVID‐19 and suggest that these GERD patients might require specific precautions such as vaccination (Vasquez‐Elera et al., 2022).

The detailed molecular mechanisms by which GERD is associated with increased risks of COVID‐19 are intricate. Previous in vivo studies have demonstrated that digestive tract played an important role in the pathogenesis of COVID‐19 since the pivotal molecules TMPRSS2 and ACE2 were found co‐expressed in both the gland and epithelial cells along with esophagus to the colon (Ng & Tilg, 2020). Moreover, based on single‐cell RNA sequencing, Xu et al. provided evidences of the potential infectivity of COVID‐19 in the digestive tract as well as the respiratory system (Zhang et al., 2020). However, the FUMA‐based genetic loci analysis was not involved in the loci of ACE2 or TMPRSS2. Conversely, the shared genetic loci between GERD and COVID‐19 were concentrated on specific transcription factor BCL11A. The colocalization analysis further validated the SNPs in BCL11A from GERD and COVID‐19 both exhibited significant correlations between GWAS and eQTL levels, implying the variants of BCL11A might participate in the potential causal effects.

B‐cell lymphoma/leukemia 11A (BCL11A) is a specialized transcription factor contributing to the development and maturation of B and T lymphopoiesis and promotes the differentiation of plasmacytoid dendritic cells (Reizis, 2019; Yu et al., 2012). Furthermore, the whole‐genome sequencing analysis has demonstrated that leucocyte differentiation induced by BCL11A is associated with progression of critical COVID‐19 (Kousathanas et al., 2022). In this study, hallmark‐based pathway enrichment analysis further indicated that 44 regulatory genes between GERD and COVID‐19 were significantly enriched in immune‐activated and inflammation‐related pathways, particularly IL‐6–JAK–STAT3 signaling, inflammatory response, TNFα signaling via NFκB and interferon‐γ response. These findings are consistent with the pathogenesis of cytokine storms in severe COVID‐19 (Ye et al., 2020). The PPI network validated the high interconnectivity among mapped regulatory molecules, emphasizing the central role of various proinflammatory cytokines, including TLR4, CXCL8, CD4, TGFB1, TNF, and IL1B.

Interestingly, the regulatory genes between GERD and COVID‐19 exhibited significant enrichment in genes upregulated in the lung, blood, and adipose tissues rather than in various gastrointestinal tissues, which suggests that the genetic predisposition of GERD primarily influences targeted tissues of COVID‐19. Additionally, extensive MR studies have also demonstrated the causal effects of obesity and body mass index on increased risks of COVID‐19 (Leong et al., 2021; Wolff et al., 2021). This supports the consistently upregulated gene expressions in adipose tissues. However, these regulatory genes were predominantly downregulated in brain tissues, implying a potential genetic susceptibility of COVID‐19 to nervous system diseases, such as Alzheimer's disease (Baranova, Cao, & Zhang, et al., 2023).

The primary strength of this study lies in the utilization of MR analysis, which is less susceptible to the causal pitfalls commonly encountered in traditional observational studies. These pitfalls often stem from confounding factors and reverse causation. Additionally, all participants included in the GWAS studies belonged to individuals of the European population, thus minimizing potential population heterogeneity and bias. However, there are still several limitations in this study. For the one, we exclusively evaluated the genetic predisposition to GERD and its potential impact on different COVID‐19 outcomes while not considering the influence of environmental factors. It is critical to note that both GERD and COVID‐19 are influenced by environmental factors, and our analysis focused solely on the genetic aspect. On the other hand, we must acknowledge that MR analyses remain susceptible to bias stemming from various forms of pleiotropy, particularly prevalent in non‐homogeneous datasets. Finally, our populations only included European ancestry from the HGI and GWAS Catalog databases, introducing potential biases and limiting the further applicability of our findings to other populations.

## 5 CONCLUSION

In conclusion, our findings not only confirm the causal effects of GERD on hospitalized and severe COVID‐19 but also provide promising insights that shared genetic loci and immune‐activated related inflammatory signaling may connect GERD with COVID‐19.

## AUTHOR CONTRIBUTIONS

Jingjing Pan contributed to data acquisition, analysis, figure presentation, drafting of the manuscript, and revision of the manuscript. Jingjing Pan and Jianhua Li contributed to the design of the study, data analysis, and manuscript revisions. All authors contributed to the article and approved the submitted version.

## CONFLICT OF INTEREST STATEMENT

The authors declare no conflicts of interest.

## CONSENT FOR PUBLICATION

Not applicable.

## Supporting information



Supporting Information

## Data Availability

The datasets that support these findings in our study are publicly available from The Covid19 Host Genetics Initiative database (https://www.covid19hg.org/results/r7/) and the GWAS Catalog database (https://www.ebi.ac.uk/gwas/home, trait ID: GCST90000514). The eQTL datasets for colocalization analysis were downloaded from eQTLGen phase I (https://www.eqtlgen.org/cis‐eqtls.html). The raw codes of this study have been expounded in corresponding R packages and GitHub tools in Section 2.
