# Supplementary material for: Gastroesophageal reflux disease increases predisposition to severe COVID‐19: Insights from integrated Mendelian randomization and genetic analysis
Source: Ann Hum Genet. 2024 Nov 12;89(1):54–65. doi: 10.1111/ahg.12584 (PMC11650540; doi:10.1111/ahg.12584)
Supplement: Supplementary file 1 — Supporting Information [file AHG-89-54-s001.docx]

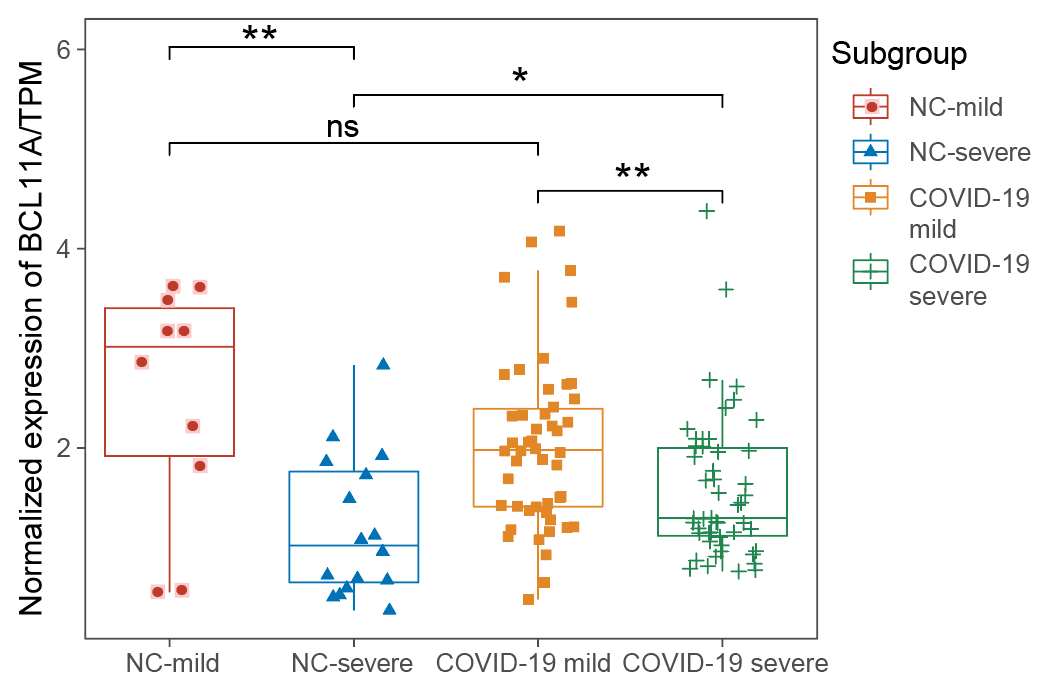


**Figure S1. Comparison of BCL11A expression between COVID-19 patients and controls at mild and severe status**. NC, normal control; TPM, transcripts per million. *, p<0.05; **, p<0.01; ns, no significance.
